# A new cluster of rhabdovirus detected in field-caught sand flies (Diptera: Psychodidae: Phlebotominae) collected from southern Thailand

**DOI:** 10.1186/s13071-021-05047-z

**Published:** 2021-11-08

**Authors:** Atchara Phumee, Supaporn Wacharapluesadee, Sininat Petcharat, Padet Siriyasatien

**Affiliations:** 1 Department of Medical Technology, School of Allied Health Sciences, Walailak University, Nakhon Si Thammarat, 80160 Thailand; 2 Research Excellence Center for Innovation and Health Products, Walailak University, Nakhon Si Thammarat, 80160 Thailand; 3 Excellent Center for Dengue and Community Public Health (EC for DACH), Walailak University, Nakhon Si Thammarat, 80160 Thailand; 4 Thai Red Cross Emerging Infectious Diseases Clinical Centre, King Chulalongkorn Memorial Hospital, Faculty of Medicine, Chulalongkorn University, Bangkok, 10330 Thailand; 5 Vector Biology and Vector Borne Disease Research Unit, Department of Parasitology, Faculty of Medicine, Chulalongkorn University, Bangkok, 10330 Thailand

**Keywords:** Molecular survey, Sand fly, *Rhabdoviridae*, Thailand

## Abstract

**Background:**

The distribution of phlebotomine sand flies is changing rapidly due to climate change. This issue has implications for the epidemiology of sand fly-borne diseases, especially sand fly-associated viruses. Few studies concerning sand fly-associated viruses have been conducted in Thailand. Therefore, this study aimed to perform a molecular survey of groups of pathogenic RNA viruses belonging to the *Orbivirus*, *Phlebovirus*, and *Flavivirus* genera and family *Rhabdoviridae* in sand fly samples collected from southern Thailand.

**Methods:**

Sand flies were collected at two locations in Trang and Songkhla provinces of southern Thailand, and individual sand fly samples were processed for species identification and virus detection. The *Orbivirus*, *Phlebovirus*, and *Flavivirus* genera and family *Rhabdoviridae* molecular determination was performed by RT-PCR, and positive samples were identified by cloning and sequencing, cell culture inoculation, and phylogenetic analysis.

**Results:**

The results presented in this study were based on the analysis of a total of 331 female sand flies. This molecular study revealed evidence of *Rhabdoviridae* family virus presence in *Phlebotomus papatasi* (3/331, 0.9%). The findings demonstrated a new cluster of rhabdovirus that was closely related to *Bactrocera dorsalis* sigmavirus strain BDSV.abc5 and the lineages of insect-specific *Rhabdoviridae*. In addition, the Bayesian tree suggested that the common ancestor of this group was the dimarhabdovirus clade. It was assumed that the virus may have switched hosts during its evolution. However, the detection of *Orbivirus*, *Phlebovirus*, and *Flavivirus* genera using specific primers for RT-PCR was negative in the collected sand flies.

**Conclusions:**

There is limited knowledge on the genetic diversity and ecology of *Rhabdoviridae* in Thailand. This is the first data regarding the circulation of *Rhabdoviridae* in *Ph. papatasi* from Thailand. We found a new cluster of rhabdoviruses that was close to the new *B. dorsalis* sigmavirus. It is possible that there is a great deal of diversity in this family yet to be discovered, and a more extensive survey for new rhabdoviruses may uncover viruses from a wide diversity of host taxa and broaden our understanding of the relationships among the *Rhabdoviridae*.

**Graphical abstract:**

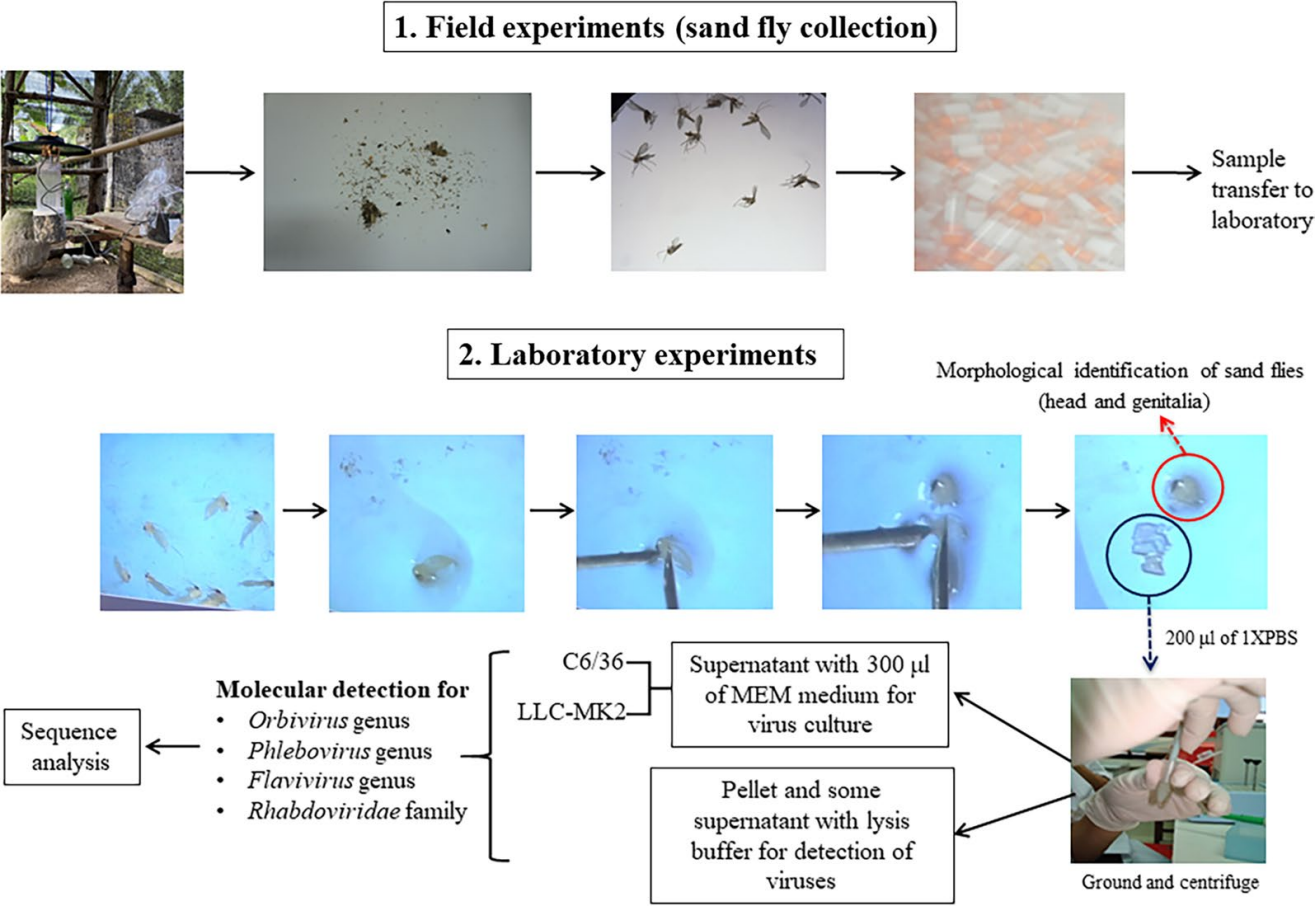

## Background

Phlebotomine sand flies (Diptera: Psychodidae) are a medically important group of insects widely distributed throughout the world. They are responsible for causing several diseases in humans and other animals, including leishmaniasis, sand fly fever, and human bartonellosis [1–3]. In southern Thailand, several sand fly-borne pathogens have been detected in various sand fly species, including *Leishmania* parasites in *Sergentomyia* (*Neophlebotomus*) *gammae*, *Se. khawi*, and *Se.* (*Parrotomyia*) *barraudi* sand flies, and *Trypanosoma* parasites in *Phlebotomus stantoni*, *Se. khawi*, *Se. indica*, *Se. anodontis*, *Ph. asperulus*, and *Ph. betisi* species [4, 5]. Sand fly fever is still a significant health problem in many regions of the world, particularly in the Mediterranean Basin, the Middle East, Africa, Central Asia, and Europe. [6, 7]. The common serotypes of phleboviruses (family *Bunyaviridae*, genus *Phlebovirus*) are sand fly fever Sicilian virus (SFSV), sand fly fever Naples virus (SFNV) and Toscana virus (TOSV), which are transmitted to humans by different species of sand flies, especially *Phlebotomus* species [8, 9]. Moreover, there are other viruses that can be transmitted by sand flies, such as family *Rhabdoviridae* [10], family *Reoviridae* [11], and family *Flaviviridae* [12]. Interestingly, rhabdoviruses can infect the cytoplasm of host cells. They possess an approximately 12-kilobase (kb) genome that usually consists of five genes (3′-N-P-M-G-L-5′) [13]. In 2015, 13 genera of rhabdoviruses were available at http://www.ictvonline.org/virusTaxonomy.asp, comprising *Cytorhabdovirus*, *Dichorhavirus*, *Ephemerovirus*, *Lyssavirus*, *Novirhabdovirus*, *Nucleorhabdovirus*, *Perhabdovirus*, *Sigmavirus*, *Sprivivirus*, *Tibrovirus*, *Tupavirus*, *Varicosavirus*, and *Vesiculovirus* [14, 15]. Similar to the majority of the known dimarhabdoviruses (e.g. genera *Sigmavirus*, *Vesiculovirus*, and *Ephemerovirus*), cytorhabdoviruses, nucleorhabdoviruses, and rhabdoviruses are carried by arthropods [16]. Despite their crucial roles in the epidemiology of the disease, in-depth knowledge of sand fly-associated viruses is still scarce. In addition, several factors associated with climate change, human activity, and different geographical regions affect sand fly-borne pathogen distribution in some parts of the world [17]. The most recent report found that the *Orbivirus* genus in *Idiophlebotomus* spp., *Phlebotomus papatasi*, and *Se. khawi* from southern Thailand was closely related to Changuinola virus (CGLV) [18]. However, it is the only report of survey studies of the distribution of sand fly-associated viruses in Thailand. Therefore, the objective of this study was to survey and screen for the presence of RNA viruses, including the genera *Orbivirus*, *Phlebovirus*, and *Flavivirus* and family *Rhabdoviridae*, in field-caught sand flies collected from the southern region of Thailand. The detection method used was reverse transcription polymerase chain reaction (RT-PCR) with the use of viral-specific primers to precisely target each virus and sequence analysis. This technique provides a rapid means for obtaining expansive results that facilitate the investigation of the phylogenetic relationships of the virus and its host. Moreover, identification of the host and virus associations remains a key challenge for virus research. The impacts of virus screening in sand flies from Thailand can offer an important contribution to the establishment of vector control programs for sand fly-associated viruses in the country and the region.

## Methods

### Sand fly collection and species identification

A total of 331 female sand flies were collected in Huai Yot District, Trang Province (7°47′6″N, 99°38′6″E), and in Na Thawi District, Songkhla Province (6°39′58.9″N, 100°42′34.6″E), Thailand, during the period 14–20 December 2020. The sand flies were collected using five to seven miniature Centers for Disease Control and Prevention (CDC) light traps, which were installed and hung from tree branches or hooks at a height of 1 m measuring from the trap hood to the ground. All traps were activated for 12 h from 6.00 p.m. to 6.00 a.m. Insects were collected from the light traps the following day. These sand flies were stored in 2-ml cryovials in liquid nitrogen and then transported to the laboratory for sorting and taxonomic identification. Individual female sand flies were placed on a sterilized slide and dissected under a stereomicroscope equipped with an ice block, using 26G  ×  1/2″ sterile needles. Head and genitalia were mounted with Hoyer’s medium on a slide, which was later used for morphological species identification based on keys proposed by Lewis [19], Phumee et al. [4], and Depaquit et al. [20]. Molecular identification was performed to confirm the sand fly species using mitochondrial cytochrome b gene (*CytB*)-PCR [21]. The abdomen and thorax of each female specimen were transferred to a sterile 1.5-ml Eppendorf tube containing 200 μl of 1× phosphate-buffered saline (PBS). All samples were then stored at −80 °C until viral screening was carried out.

### Viral RNA extraction and viral RNA screening

All sand fly samples were ground in 200 μl of 1× PBS and then centrifuged at 11,000×*g* for 5 min. Subsequently, 100 μl of supernatant with 300 μl of minimum essential medium (MEM) was used for virus culture. The pellet and some of the supernatant with lysis buffer were processed for viral RNA extraction using the Invisorb Spin Virus RNA Mini Kit (STRATEC Molecular GmbH, Germany) following the manufacturer’s instructions. The extracted RNA samples were immediately used for detection of virus families, and the remaining samples were stored at −80 °C. The RNA samples were amplified for the detection of the *Orbivirus* [22], *Phlebovirus* [23], and *Flavivirus* [24] genera and family *Rhabdoviridae* [25]. All the methods for viral RNA detection were briefly described in Phumee et al. [18]. PCR amplicon sizes were determined by 1.5% ultrapure low-melting-point (LMP) agarose (Gibco, USA) gels, and gels were stained with ethidium bromide and visualized with Quantity One quantification analysis version 4.5.2 software (Gel Doc EQ System; Bio-Rad, USA). The estimated sizes for the *Orbivirus*, *Phlebovirus*, and *Flavivirus* genera and family *Rhabdoviridae* were 188, 244, 270, and 460 base pairs (bp), respectively. A synthesized plasmid was used as a positive control to prevent sample contamination.

### Virus isolation

One hundred microliters of each virus-positive suspension was inoculated onto confluent monolayers of *Aedes albopictus* C6/36 (ATCC CRL-1660) or rhesus monkey kidney epithelial (LLC-MK2) (ATCC CCL-7) cells for 1 h in 24-well plates grown in MEM (Gibco, USA) supplemented with 10% heat-inactivated fetal bovine serum (FBS) (Gibco, USA), 100 U/ml penicillin, and 100 μg/ml streptomycin (Gibco, USA). The cultures were incubated at 28 °C (C6/36) and 37 °C (LLC-MK2) in 5% CO_2_. Cytopathic effects (CPEs) were monitored and observed daily for the following 6–10 days. The positive isolates were continually propagated in C6/36 cells and LLC-MK2 cells grown in MEM supplemented with 10% heat-inactivated FBS (Gibco, USA) at 28 °C (C6/36) and 37 °C (LLC-MK2) in a 5% CO_2_ environment for additional passages. The culture supernatants were harvested for identification and sequencing. Uninfected C6/36 or LLC-MK2 cells were used as negative controls.

### Cloning and sequencing

PCR products were either directly sequenced or cloned into the pGEM-T Easy Vector (Promega, USA), transformed into DH5α competent cells, and screened by a blue–white colony selection system. The suspected positive colonies were cultured and used for further plasmid DNA extraction using the Invisorb Spin Plasmid Mini kit (STRATEC Molecular GmbH, Germany) following the manufacturer’s instructions. Purified plasmids were sent for sequencing by 1st BASE DNA sequencing services (1st BASE Laboratories, Malaysia) using universal forward T7 primers. Several independent PCR clones (3–5 clones) were analyzed to produce a consensus nucleotide sequence for each virus. Because of genetic variation within the degenerate primer sequences, they were excluded from the analysis. Nucleotide sequences were analyzed by comparing them with the reference viral sequence in the GenBank database using the BLASTn tool in the BLAST program. This tool searches nucleotide databases using percentage nucleotide queries and identity.

### Phylogenetic analysis

The nucleotide sequences were aligned using the BioEdit version 7.2.5 sequence alignment editor [26] and MAFFT (multiple alignment using fast Fourier transform) version 7 software (https://mafft.cbrc.jp/alignment/software/) [27]. Phylogenetic trees of viruses were generated using both maximum likelihood (ML) and Bayesian approaches with IQ-TREE on the IQ-TREE web server (http://iqtree.cibiv.univie.ac.at/) with 1000 ultrafast bootstrap replicates. The best-fit model of substitution was identified using the auto function on the IQ-TREE web server [28]. Finally, the phylogenetic tree was viewed and edited with FigTree version 1.4.4 (http://tree.bio.ed.ac.uk/software/figtree/).

### Statistical analysis

This study is a descriptive analysis. The sample size was calculated following a simple formula [29] by estimated prevalence as a percentage from a pilot study and a previously published paper from our team [18], with 95% confidence. The data from the sand fly survey were calculated as the total number of sand fly species divided by the total population of the total number of all sand fly samples. The percentage of virus-sand fly infection was also calculated for the virus-positive sand flies, which was calculated by dividing the total number of positive samples of sand flies by the total number of all sand fly samples. All statistical analyses were performed using Microsoft Excel 2019 (Microsoft Corporation, USA).

## Results

**Fig. 1:**
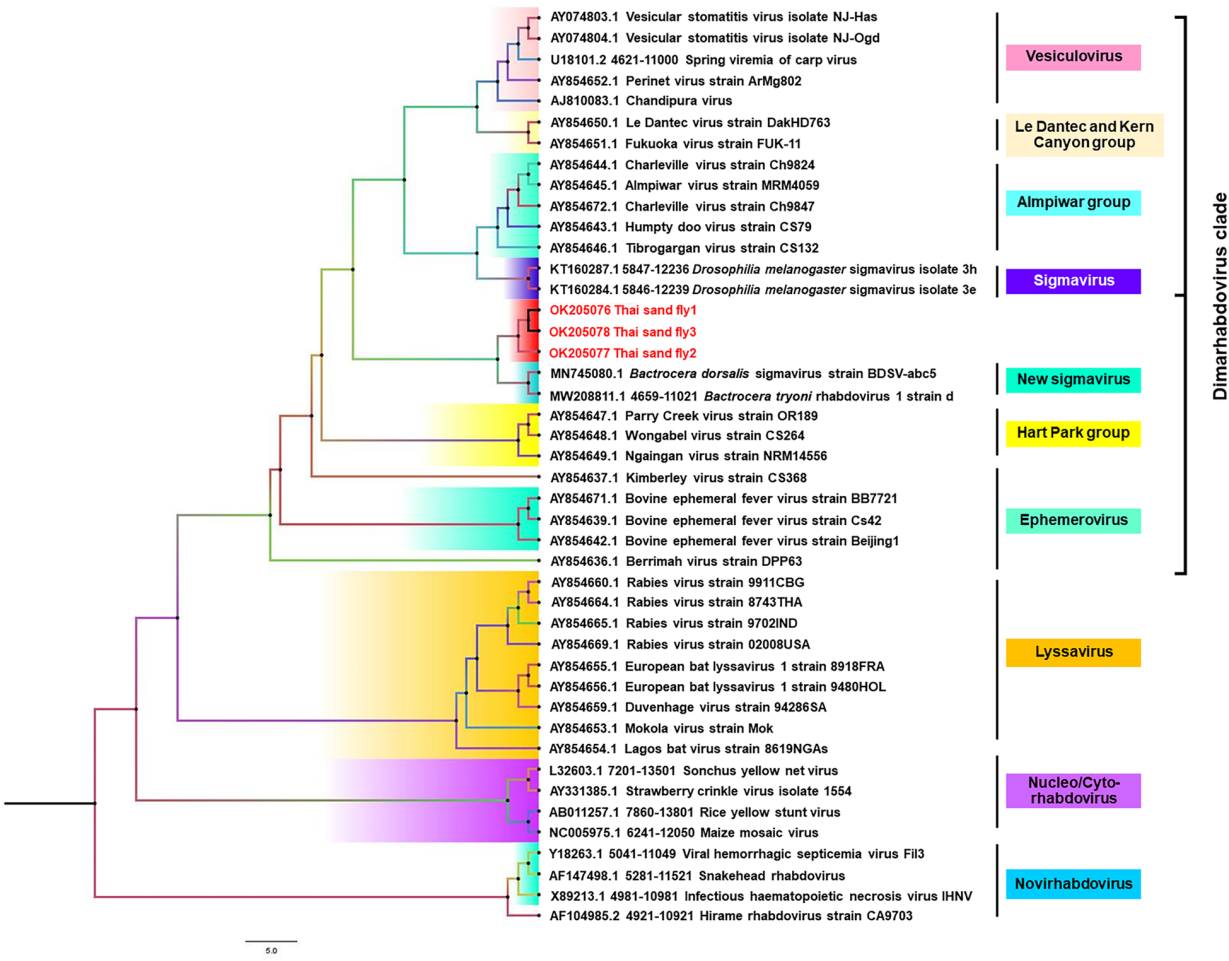
Phylogeny of the *Rhabdoviridae* from partial nucleotide sequences of the L gene. The tree was constructed with IQ-TREE using maximum-likelihood bootstrap support (1000 replicates). The best-fit model of substitution was found using the auto function on the IQ-TREE web server. The sequences described in this study are indicated in red

**Fig. 2:**
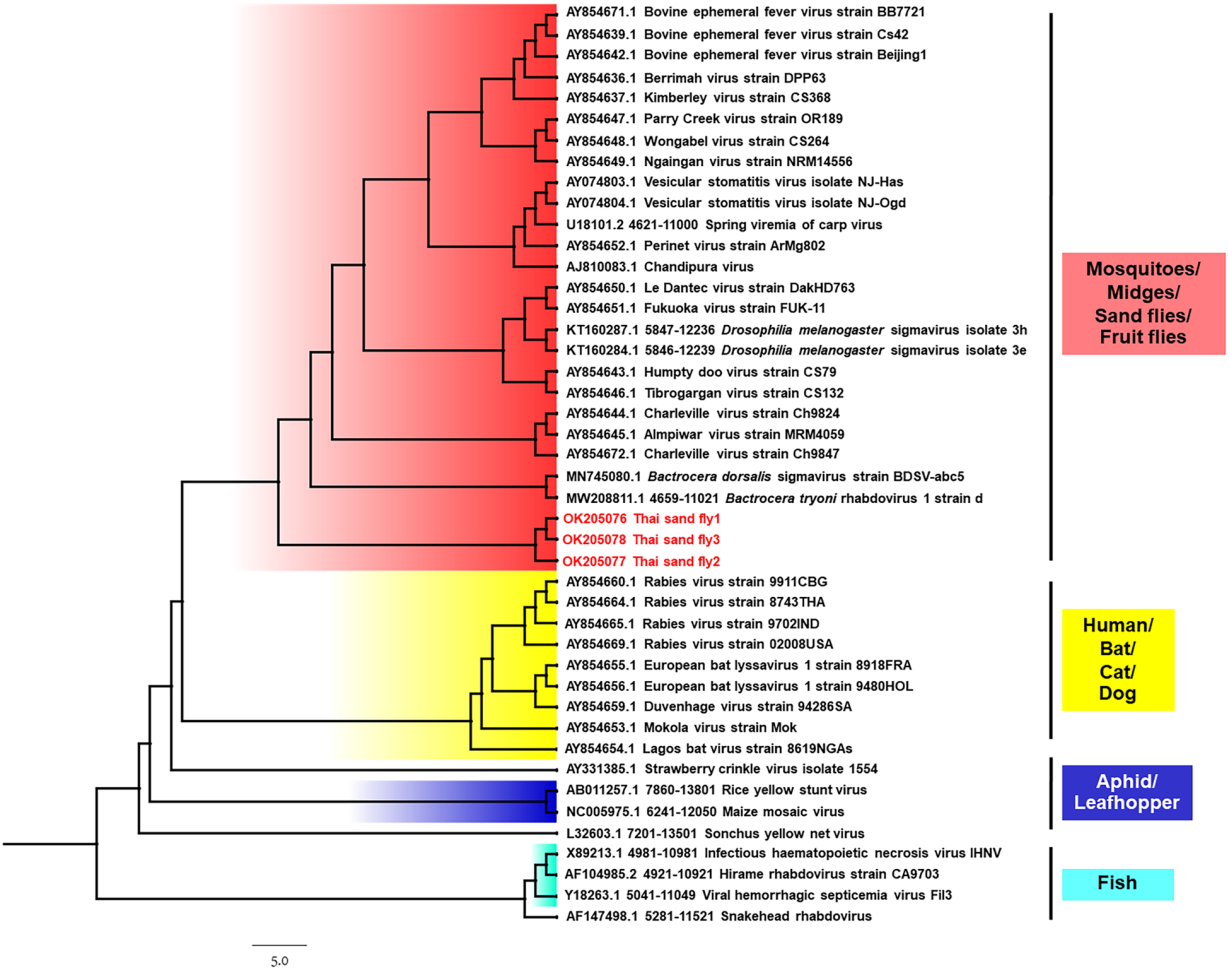
Bayesian nucleotide tree of partial sequence alignments for insect-specific *Rhabdoviridae* using IQ-TREE. The sequences described in this study are indicated in red, and their origin (insect) is also indicated

A total of 331 specimens were analyzed. The samples included 162 (48.9%) sand flies collected in Trang and 169 (51.1%) sand flies collected in Songkhla. *Phlebotomus stantoni* and *Se. indica* were the most abundant sand fly species found in Trang and Songkhla provinces, respectively. *Idiophlebotomus* spp., *Ph. mascomai*, and *Ph. papatasi* were found only in Trang province. All samples were screened for viruses: *Orbivirus*, *Phlebovirus*, and *Flavivirus* genera and family *Rhabdoviridae*. The *Orbivirus*, *Phlebovirus*, and *Flavivirus* genera RT-PCRs were negative in all sand fly samples (Table 1). Interestingly, family *Rhabdoviridae* RNA was detected in three (0.9%) *Ph. papatasi* samples. Viral isolation was not successful in cell culture inoculation of the positive samples. No cytopathic effect (CPE) was observed in three positive sand fly samples, and culture supernatants remained negative for the family *Rhabdoviridae*. *Rhabdoviridae* family RNA was detected in three samples of *Ph. papatasi*. Sequencing of the L-region amplicon revealed a sequence length of approximately 450 bp. Sequences of all three samples were deposited in GenBank (accession nos. OK205076–OK205078). We observed that these three sequences had similarity of 74.16% compared to *Bactrocera dorsalis *sigmavirus strain BDSV.abc5 (accession no. MN745080), which has been reported as a new sigmavirus in the family *Rhabdoviridae* from fruit flies. The intramural diversity of three sequences and other sigmaviruses showed 19.2–41.5% nucleotide similarity. The ML analysis using the sequences from the three positives of family *Rhabdoviridae* in this study revealed a separate clustering from *B. dorsalis *sigmavirus strain BDSV.abc5 (accession no. MN745080), *B. tryoni* rhabdovirus 1 strain d (accession no. MW208811), rhabdoviruses or dimarhabdoviruses supergroup; vesiculovirus, Le Dantec and Kern Canyon group, Almpiwar group, sigmavirus, Hart Park group, and ephemerovirus (Fig. 1). Additionally, we conducted Bayesian analysis to investigate the correlation between the viruses and their host evolution by combining phylogenetic data with their host information. The results revealed that these three new sequences of sigmaviruses in *Ph. papatasi* from Thailand were categorized in a well-supported monophyletic group that was distinct from other dimarhabdoviruses (Fig. 2).

**Table 1:** Number of detected sand fly species and viruses

Province	Sand fly species	No. of sand flies	No. of positive virus samples			
			*Orbivirus*	*Phlebovirus*	*Flavivirus*	*Rhabdoviridae*
Trang	*Idiophlebotomus *spp.	15	0	0	0	0
	*Phlebotomus stantoni*	37	0	0	0	0
	*Ph. mascomai*	18	0	0	0	0
	*Ph. papatasi*	16	0	0	0	3
	*Sergentomyia hivernus*	27	0	0	0	0
	*Se. indica*	21	0	0	0	0
	*Se. iyengari*	9	0	0	0	0
	*Se. khawi*	19	0	0	0	0
Songkhla	*Ph. stantoni*	24	0	0	0	0
	*Se. barraudi*	39	0	0	0	0
	*Se. hivernus*	24	0	0	0	0
	*Se. indica*	45	0	0	0	0
	*Se. khawi*	37	0	0	0	0
Total		331	0	0	0	3

## Discussion

The study of pathogens in arthropods is an important part of surveillance programs. It can provide data about circulating pathogens and their vectors, assess potential public health threats, predict probable disease emergence in susceptible humans or animals, and establish optimal diagnostics and interventions to prevent disease transmission [30, 31]. In the present study, we detected family *Rhabdoviridae* RNA in three (0.9%) samples of *Ph. papatasi* sand flies from Trang Province. The *Ph. papatasi* was not found in Songkhla Province in this study. However, the results obtained did not reveal the circulation of the *Orbivirus*, *Phlebovirus*, or *Flavivirus* genera in field-caught sand flies. To the best of our knowledge, this is the first study undertaken to identify a new cluster of rhabdoviruses from *Ph. papatasi* in Thailand that was genetically closely related to *B. dorsalis * sigmavirus strain BDSV.abc5. Rhabdoviruses, a family of negative-sense RNA viruses (*Mononegavirales*: *Rhabdoviridae*), are a large and ecologically diverse group of viruses [32]. For the ML phylogenetic tree, the sequences from this study showed separate clustering, exhibiting a close relationship to *B. dorsalis* sigmavirus strain BDSV.abc5 of the oriental fruit fly (accession no. MN745080) [33] and *B. tryoni *rhabdovirus 1 strain d of a tephritid fruit fly (accession no. MW208811) [34]. The viruses have been reported as new sigmaviruses in the family *Rhabdoviridae* [33, 34]. *Sigmavirus* genera infects a variety of fruit flies, including *Drosophila melanogaster*, *D. immigrans*, and *D. ananassae* [35]. It was found that flies infected with the sigmavirus of *D. melanogaster* (DMelSV) became paralyzed and died upon exposure to CO_2_, whereas uninfected flies recovered after exposure to CO_2_ [36]. In addition, rhabdoviruses are involved in paralysis by binding to acetylcholine receptors on the nervous tissue of flies [37]. Several reports suggested that rhabdovirus injection in aphids [38], *Culex* mosquitoes [39], and *Drosophila* [37] reduced their lifespan after CO_2_ exposure. From the aforementioned discussion, it is postulated that rhabdoviruses may exist in common groups of insects and may serve as common pathogens in those insect populations. Our findings demonstrated the first use of molecular techniques to detect the family *Rhabdoviridae* in sand flies from southern Thailand. The results revealed the presence of a new cluster of rhabdoviruses, which was closely related to the new sigmavirus in *Ph. papatasi*. The phylogeny of these sequences was reconstructed using Bayesian analysis with amino acid sequences and models of protein evolution. Our phylogeny showed that new sigmaviruses in *Ph. papatasi* are in the same lineage of dimarhabdoviruses, which suggested that infecting arthropod viruses emerged from this clade and might have undergone host switching during its evolution rather than cospeciation with them. Previous studies have revealed that for a pathogen to infect a new host species, it must adapt and adjust to the new environment in which it settles [40–42]. Additionally, the phylogenetic distance and root sharing between hosts also elucidate the diversity in virulence after cross-species transmission. Virulence increases when viruses jump between more distantly related hosts [43, 44]. Here, we report for the first time a new cluster of rhabdoviruses closely related to the new sigmavirus in sand flies from Thailand. Zhang et al. [33] implied that there was a strong association between sigmaviruses and their hosts. Therefore, the discovery of this virus may enrich our understanding of the diversity of RNA viruses in sand flies and provide information about virus association with their host and their adaptation in insects. This new sigmavirus may be a general rhabdovirus that is unlikely to cause high mortality. Unfortunately, the isolation of the virus in this study in *Ae. albopictus* C6/36 (ATCC CRL-1660) and rhesus monkey kidney epithelial (LLC-MK2) (ATCC CCL-7) cells was not successful, and the RT-PCR performed with the infected cells was negative. This likely resulted from a combination of factors associated with the C6/36 and LLC-MK2. Indeed, C6/36 cells have been used extensively for the isolation of rhabdoviruses [45, 46], but they may not be permissive to all insect viruses. In this regard, a non-specific cell line should be used instead. This may indicate that the culture of C6/36 or LLC-MK2 cells, although convenient, is be ideal. Few studies have shown different culture results from different cell types. A previous report in Italy showed the successful isolation of a novel rhabdovirus from an insectivorous bat (*Pipistrellus kuhlii*) in Vero cells [47]. Another study showed that infecting LL-5 cells (sand fly cell line derived from sand fly *Lutzomyia longipalpis*) with vesicular stomatitis virus (VSV) from a sand fly could establish productive, long-lasting persistent infections. However, sustained persistence was difficult in C6/36 cells in the study [48–50]. Extensive studies aimed at identifying insect rhabdovirus vectors and their distribution in nature could help us understand the natural evolution of this virus; moreover, extensive surveys to demonstrate its presence in its natural vectors or host may have benefit in terms of demonstrating early signals of its circulation before emergence of diseases from such viruses and sand flies. Furthermore, the technical limitations of the present study based on conventional RT–PCR for viral screening, high-throughput experimental approaches, or metagenomic experimental design combined with the use of next-generation sequencing methods should be addressed in future studies.

## Conclusions

This study reported a new cluster of rhabdoviruses closely related to the new sigmavirus in sand flies from southern Thailand. Additionally, the results also provide information on virus association with insect host adaptation. Although rhabdoviruses are already considered common pathogens in terms of insect-specific viruses, extensive surveys and comprehensive studies of new rhabdoviruses might be able to clarify and demonstrate the wide diversity of their host taxa as well as epidemiological data of this virus in sand flies. Moreover, further studies will be able to provide a better understanding of the relationships between rhabdoviruses and sand flies in Thailand.

## Data Availability

The datasets used and/or analyzed during the current study are available from the corresponding author on reasonable request.

## Abbreviations

bp: Base pairs
CPE: Cytopathic effect
PCR: Polymerase chain reaction
RT-PCR: Reverse transcriptase PCR
RNA: Ribonucleic acid
